# Wheat‐Dependent Exercise‐Induced Anaphylaxis Mimicking Acute Asthma in an Adolescent: A Case Report

**DOI:** 10.1155/crii/3365796

**Published:** 2026-09-22

**Authors:** Xia Zhao, HongBin Song, Hui Li, Ying Yan, Ping Li, Bing Zhuan, Wei Zhou

**Affiliations:** ^1^ Department of Respiratory Medicine, People’s Hospital of Ningxia Hui Autonomous Region, Ningxia Medical University, Yinchuan 750031, Ningxia Hui Autonomous Region, China, nxmu.edu.cn; ^2^ Department of Dermatology, People’s Hospital of Ningxia Hui Autonomous Region, Ningxia Medical University, Yinchuan 750031, Ningxia Hui Autonomous Region, China, nxmu.edu.cn

**Keywords:** adolescent, food allergy, wheat, wheat-dependent exercise-induced anaphylaxis

## Abstract

**Background:**

Wheat‐dependent exercise‐induced anaphylaxis (WDEIA) is an acute immediate hypersensitivity reaction triggered by exercise following the ingestion of wheat products, characterized by severe manifestations including urticaria, angioedema, dyspnea, hypotension, and even anaphylactic shock. Notably, WDEIA is rarely induced by wheat ingestion alone or exercise alone, and it is frequently misdiagnosed as asthma due to insufficient clinical awareness, leading to recurrent episodes.

**Case Presentation:**

We report an 18‐year‐old male patient who developed dyspnea, laryngeal tightness, generalized urticaria, facial edema, and hypotensive shock shortly after exercise following wheat‐based food consumption. The patient was initially misdiagnosed with asthma at a local hospital and achieved symptom remission after emergency treatment with epinephrine and dexamethasone. WDEIA was clinically diagnosed based on the clinical history, serum total immunoglobulin E (IgE) levels, wheat‐specific IgE (sIgE) assays, and allergen skin prick tests (SPTs). Different from previously reported WDEIA cases with relatively single clinical presentations, this patient had a long‐term atopic diathesis accompanied by multiple allergic sensitizations, which increased the complexity and atypicality of clinical manifestations. The patient was instructed to exercise on an empty stomach or refrain from wheat intake for 4–6 h before exercise. No recurrent allergic episodes occurred during the 5‐month follow‐up period, which verified the effectiveness of standardized preventive strategies.

**Conclusion:**

This case highlights that WDEIA should be considered in the differential diagnosis of acute asthma exacerbation or exercise‐related allergic reactions, especially in young atopic individuals. Early identification, prompt administration of epinephrine, and strict avoidance of wheat products before exercise are critical for the effective management and prevention of WDEIA. Combined with a literature review, this study summarizes the pathogenesis, clinical features, diagnostic approaches, emergency management, and preventive strategies of WDEIA, aiming to enhance clinicians’ ability to recognize, diagnose, and manage this disease.

## 1. Introduction

Food‐dependent exercise‐induced anaphylaxis (FDEIA) represents a unique subtype of food allergy that is specifically triggered by physical exercise. Its typical clinical manifestations encompass generalized urticaria and life‐threatening allergic reactions, including hypotension and anaphylactic shock [1]. FDEIA can be provoked by a wide spectrum of food allergens, among which wheat has been well‐documented as the most prevalent causative trigger. Correspondingly, wheat‐dependent exercise‐induced anaphylaxis (WDEIA) is recognized as the most common variant of FDEIA. WDEIA is a rare but potentially life‐threatening immunoglobulin E (IgE)‐mediated food allergy, with a diverse clinical spectrum ranging from urticaria and angioedema to respiratory distress, gastrointestinal disturbances, hypotension, and severe anaphylactic shock. These acute allergic episodes predominantly occur when physical activity is performed within 1–6 h following wheat ingestion [2]. Notably, in addition to physical exercise, multiple cofactors—such as aspirin use, alcohol consumption, active infection, psychological stress, and menstruation—have been confirmed to trigger immediate hypersensitivity reactions following wheat exposure [3]. Nevertheless, both patients and clinicians generally lack sufficient awareness and clinical proficiency regarding this condition, which inevitably leads to substantial diagnostic delays. Such prolonged misdiagnosis frequently results in recurrent allergic attacks in affected individuals, and critical cases may even progress to life‐threatening emergencies due to a missed definitive diagnosis. Given the above clinical gaps, we herein report a clinically diagnosed case of WDEIA in a patient admitted to our hospital, whose initial presentation mimicked acute asthma. The present case report aims to systematically elaborate the clinical characteristics, core diagnostic criteria, differential diagnostic essentials, and preventive strategies of WDEIA, with the ultimate goal of enhancing clinicians’ overall understanding and vigilance toward this underrecognized, life‐threatening allergic disorder.

## 2. Case Presentation

An 18‐year‐old male student was admitted to our hospital on October 29, 2025, with a 21‐h history of acute dyspnea and a cutaneous rash of sudden onset following physical exercise. His clinical course, past medical history, laboratory findings, definitive diagnosis, and follow‐up outcomes are described in detail below.

At 17:50 on October 28, 2025, the patient consumed a chicken skeleton with wheat noodles. At 19:00, he engaged in indoor badminton; half‐an‐hour after the exercise, he suddenly developed dyspnea and a sensation of laryngeal tightness. Initially, there was no loss of consciousness, skin rash, cough, or sputum production, and no other discomfort was reported. He immediately ceased exercise, but his symptoms failed to improve after 10 min of rest.

After resting in an outdoor staircase for an additional 5 min, he developed a mild cough, accompanied by nausea and vomiting (vomitus consisted of gastric contents). His dyspnea was slightly relieved postemesis; however, severe dyspnea recurred within the subsequent 5 min, accompanied by perioral and facial numbness, generalized tremor, diffuse pruritus, urticarial rashes on the medial aspects of both forearms and the posterior back, facial edema, dry throat, sore throat, pharyngeal foreign body sensation, and dizziness. No disturbance of consciousness, syncope, chills, fever, nasal congestion, nasal itching, rhinorrhea, abdominal pain, or diarrhea was noted during this episode. The patient did not take any medications, and his friends promptly called the emergency medical service (120).

Upon the arrival of emergency medical personnel (120), the patient’s vital signs were recorded as follows: blood pressure 80/40 mmHg, heart rate 80 beats per minute, and pulse oxygen saturation 90%. Auscultation of both lungs revealed extensive wheezing rales. Immediately, 0.5 mg of epinephrine was administered intramuscularly, and 10 mg of dexamethasone sodium phosphate was given intravenously; the patient was then urgently transferred to the emergency department of a local hospital. Routine blood tests, as well as liver and renal function examinations, showed no significant abnormalities. The initial diagnosis was anaphylactic shock and bronchial asthma. Symptomatic treatments were administered, including calcium gluconate for antiallergy, diprophylline for antiasthma, and fluid replacement. Half‐an‐hour later, the patient’s symptoms improved significantly, and he was discharged after an uneventful observation period. Subsequently, he was presented to our hospital for further diagnosis and standardized management.

Past medical history: The patient had a 16‐year history of allergic rhinitis, which was exacerbated in spring and autumn or upon exposure to irritant odors; he took oral loratadine intermittently for symptomatic relief. He had a history of urticaria in childhood and denied any known food or drug allergies.

After admission, relevant laboratory and auxiliary examinations were performed: routine blood test showed a normal eosinophil count (0.10 × 10^9^/L); total serum IgE level was 480 IU/mL; specific IgE (sIgE) to wheat was 1.47 KUA/L (Grade 2). Chest computed tomography (CT), pulmonary function tests, and fractional exhaled nitric oxide detection revealed no abnormal findings.

Based on the patient’s clinical manifestations and laboratory examination results, the patient was clinically diagnosed with WDEIA. The patient was advised to exercise on an empty stomach or refrain from consuming wheat products within 4–6 h prior to exercise. At the 5‐month follow‐up, the patient reported no recurrence of allergic symptoms after strictly adhering to the advice of avoiding wheat intake before exercise.

Comprehensive skin prick testing for inhalant and food allergens was undertaken with commercial extracts for routine allergens. Allergen prick‐test solutions, positive‐control solution (54.6 mmol/L histamine phosphate), and negative‐control prick‐test solution (isotonic saline) were supplied by Xinhualian‐Union Pharmaceutical, Beijing, China. For gluten, the prick‐to‐prick method was applied using freshly prepared wet gluten obtained by washing dough made from commercial wheat flour (*Triticum aestivum*) to remove starch; the gluten was assayed immediately without freezing or storage. Skin prick tests (SPTs) were performed using standard lancets following standard prick‐test operating procedures. SPT results, serum total IgE, and wheat‐sIgE values were recorded, and the test results are shown in Table 1.

**Table 1:** Laboratory findings of the reported case.

Category	Test item	Result	Reference range/positivity criterion
Serum IgE	Total serum IgE	480 IU/mL	0–100 IU/mL
	Wheat‐specific IgE	1.47 kUA/L	<0.35 kUA/L
SPT	Negative control Positive control Commercial wheat extract	0 mm MWD 3.5 mm MWD ++, 4 mm MWD	–^a^
	Wheat wet‐gluten^b^	++, 6 mm MWD	
	Poplar pollen	+++, 10 mm MWD	
	Willow pollen	++++, 11 mm MWD	
	London plane pollen Elm pollen	++++, 11 mm MWD +++, 8 mm MWD	
	*Sabina chinensis pollen*	++, 5 mm MWD	
	*Artemisia sieversiana*	++++, 16 mm MWD	
	*Artemisia annua* *Humulus scandens*	++++, 13 mm MWD ++, 5 mm MWD	

*Note:* SPT results were evaluated 15–20 min after puncture, and MWD was calculated by measuring two perpendicular wheal diameters.

Abbreviations: IgE, immunoglobulin E; MWD, mean wheal diameter; SPT, skin prick test.

^a^A positive skin prick test reaction was defined as a MWD at least 3 mm greater than that of the simultaneous negative diluent control [4]. A widely adopted clinical grading scale was used to categorize the severity of cutaneous responses [5]: (−) negative (no increased wheal or erythema); (+) weakly positive (2–4 mm wheal with erythema); (++) moderately positive (4–7 mm wheal with erythema); (+++) strongly positive (7–10 mm wheal with marked erythema); (++++) very strongly positive (>10 mm wheal with extensive erythema and/or pseudopods). Only positive SPT reactions are listed in this table.

^b^Tested by the prick‐to‐prick method.

## 3. Discussion

WDEIA is a distinct subtype of IgE‐mediated wheat allergy. Patients typically present with symptoms such as urticaria, angioedema, dyspnea, hypotension, syncope, and shock during physical exercise within hours of wheat ingestion, while isolated wheat consumption or exercise alone rarely triggers such reactions. ω‐5 gliadin and high‐molecular‐weight glutenin subunits are recognized as the major allergens of WDEIA [6].

The clinical diagnosis of WDEIA relies on the comprehensive integration of clinical history, SPTs, and serum‐sIgE detection. Serum ω‐5‐gliadin‐sIgE can support the diagnosis of WDEIA [7], although it was not measured in this patient. For patients with unidentified allergens or inconsistent laboratory and clinical manifestations, food–exercise provocation testing serves as an important supplementary diagnostic modality and is widely regarded as the diagnostic gold standard. However, this testing method possesses prominent inherent limitations. Its results are susceptible to multiple confounding variables, including exercise intensity, ambient temperature, and humidity, leading to poor test reproducibility. A negative provocation result cannot completely rule out WDEIA, which may stem from inappropriate food dosages, insufficient cofactor exposure, or nonstandardized exercise protocols. Furthermore, no unified global operational guidelines have been established for this test, and its cumbersome procedures and stringent environmental control requirements further increase the result variability. Most critically, provocation testing may trigger life‐threatening systemic anaphylaxis [8–10]. As one study documented, a patient with WDEIA developed hypotensive anaphylaxis during the challenge protocol and required intravenous epinephrine [11]; hence, the whole procedure must be conducted under continuous supervision by trained medical staff.

This case report describes an adolescent patient with WDEIA who has resided in northern China for a long time. Wheat‐derived foods such as noodles, steamed buns, and pancakes are staple diets for local residents. The patient had never experienced allergic reactions after ingesting wheat products previously. On this occasion, he developed anaphylactic manifestations, including dyspnea, urticaria, gastrointestinal symptoms, and hypotension after physical exercise following wheat intake. Comprehensive SPTs yielded positive results for wheat and gluten. Serum‐sIgE to wheat was measured at 1.47 KUA/L (Grade 2). The patient was instructed to avoid wheat consumption on an empty stomach or within 4–6 h prior to exercise. No recurrent episodes were observed during the 5‐month follow‐up, which was consistent with the clinical diagnostic criteria for WDEIA [2].

The clinical manifestations of WDEIA include cutaneous symptoms such as urticaria and angioedema, respiratory symptoms such as dyspnea and bronchospasm, gastrointestinal symptoms such as abdominal pain, diarrhea, and vomiting, as well as cardiovascular symptoms such as hypotension, dizziness, and even syncope [12]. WDEIA is the result of the combined action of food and exercise, and its pathogenesis is not yet fully clear. Relevant studies have shown that strenuous exercise can cause a redistribution of blood flow in the human body, increasing blood flow and velocity in exercising organs such as muscles, which in turn leads to relative intestinal mucosal ischemia. Exercise also reduces gastric acid secretion, affects the normal digestive process of food, and causes insufficiently degraded allergenic proteins (such as gliadin) in food to be absorbed into the blood, inducing the body to produce sIgE. When the body ingests the same type of food again, the allergenic proteins can bind to circulating sIgE, activate mast cells, and promote their degranulation, releasing a large number of inflammatory mediators such as histamine. In addition, acidic metabolites such as lactic acid produced by strenuous exercise can lower blood pH, further enhancing the activity of mast cells; at the same time, exercise can cause an increase in body temperature, which also promotes inflammatory cells such as mast cells to release mediators, ultimately leading to a series of allergy‐related clinical symptoms [13, 14].

The patient initially presented with dyspnea and wheezing that closely mimicked an acute asthma exacerbation, making clinical misdiagnosis highly probable. However, the patient also developed multisystem allergic reactions outside the respiratory system. Chest CT, pulmonary function tests, and fractional exhaled nitric oxide measurements showed no abnormalities. In addition, the patient’s symptoms were strongly associated with wheat ingestion and subsequent exercise, which is not a typical trigger for asthma. These clinical features facilitate the differentiation between WDEIA and bronchial asthma, thereby avoiding misdiagnosis.

Studies have shown that 59.7% of FDEIA patients also suffer from other atopic diseases, the most common of which are allergic rhinitis, asthma, allergic conjunctivitis, and atopic dermatitis [15]. In addition, 37.2% of patients have a history of urticaria, with chronic spontaneous urticaria being the most common subtype [15]. From a pathophysiological perspective, mast cells serve as key effector cells in both food allergy and urticaria. Although recent studies have found mast cell subsets with different functions, the potential common mechanisms or pathways between these two diseases still need further exploration [10].

It is worth noting that the patient in this case had a history of urticaria in childhood and has long suffered from allergic rhinitis. This test showed that his total IgE level was significantly elevated, and he had strong positive reactions to multiple inhalant and food allergens. These clinical manifestations suggest that the patient has a severe atopic diathesis, which is a potential risk factor for WDEIA and may increase the risk of severe anaphylaxis. This case further confirms the correlation between atopic diathesis and the occurrence of WDEIA.

Timely first‐aid measures are particularly critical during acute episodes of WDEIA. The preferred treatment is an immediate intramuscular injection of epinephrine, which is crucial for reversing anaphylactic shock [16]. In this case, the medical staff administered intramuscular epinephrine to the patient in a timely manner, combined with intravenous injection of dexamethasone, which quickly relieved the patient’s discomfort and effectively prevented the disease from developing in a more severe direction. For WDEIA patients, the focus of management is to avoid eating wheat products before exercise and to identify other important enhancing factors that are important for the individual [17]. In this case, we advised the patient to choose fasting exercise or avoid eating any wheat products within 4–6 h before exercise. No recurrence was reported during the 5‐month follow‐up, while the patient avoided wheat intake before exercise. Patients should carry an epinephrine autoinjector with them at all times. In the event of an accidental episode, they can perform self‐rescue in a timely manner to gain time for subsequent medical treatment. In addition, the patient’s own health management is also indispensable. They need to actively understand the pathogenesis of WDEIA, the factors that may trigger episodes, and specific prevention methods, learn to identify early signs of allergic reactions, and proficiently master basic first‐aid skills. In this case, the patient promptly stopped exercising and actively sought help after the onset, which is a correct approach that won valuable time for subsequent effective treatment.

At present, the clinical treatment of WDEIA mainly focuses on avoiding triggers and first aid during acute episodes, and there is no radical treatment targeting the etiology. In the future, we need to further explore new treatment directions, such as immunotherapy.

This case highlights that WDEIA should be considered in the differential diagnosis of acute asthma exacerbation or exercise‐related allergic reactions, especially in young atopic individuals with a history of allergic diseases. Early identification of WDEIA, prompt administration of epinephrine during acute episodes, and strict avoidance of wheat products 4–6 h prior to exercise are critical for the effective management and prevention of recurrent episodes. Combined with a literature review, this study summarizes the key pathogenesis, typical clinical features, standardized diagnostic approaches, emergency management measures, and practical preventive strategies of WDEIA, which is expected to enhance clinicians’ ability to recognize, diagnose, and manage this underrecognized, life‐threatening allergic disorder, thereby reducing the incidence of misdiagnosis and improving patient prognosis.

This study has several limitations. First, neither wheat‐cofactor provocation nor serum ω‐5‐gliadin‐sIgE testing was performed. Thus, WDEIA was clinically diagnosed relying on clinical history, total serum IgE, wheat‐sIgE, and prick‐to‐prick skin‐test findings instead of definitive provocation confirmation. Second, fresh wet gluten for prick‐to‐prick testing was homemade from commercial wheat flour. While this approach enhances sensitivity for suspected WDEIA, the in‐house‐prepared reagent lacks standardization and is not interchangeable with commercial gluten or gliadin extracts; raw‐material variability and manual preparation may affect test performance [18]. Third, this is a single‐case report, limiting the generalizability of our observations. Larger‐scale studies are therefore needed.

This case report was conducted in accordance with the ethical principles of the Declaration of Helsinki. Written informed consent was obtained from the patient and his legal guardians before manuscript submission and publication. All identifiable personal information in this article has been completely anonymized to protect patient privacy. No ethical approval was required for this individual case report according to the institutional ethical guidelines.

## Funding

This study was funded by the Ningxia DST (Grants 2025GKLRLX19 and 2024BEG02013).

## Conflicts of Interest

The authors declare no conflicts of interest.

## Data Availability

The data that support the findings of this study are available from the corresponding author upon reasonable request.
